# The Effect of Multi-Layer Stacking Sequence of TiO_x_ Active Layers on the Resistive-Switching Characteristics of Memristor Devices

**DOI:** 10.3390/mi11020154

**Published:** 2020-01-30

**Authors:** Minho Kim, Kungsang Yoo, Seong-Pil Jeon, Sung Kyu Park, Yong-Hoon Kim

**Affiliations:** 1 School of Advanced Materials Science and Engineering, Sungkyunkwan University, Suwon 16419, Korea; mzaq21cx@naver.com (M.K.); kunsangyoo@naver.com (K.Y.); 2 School of Electrical and Electronic Engineering, Chung-Ang University, Seoul 06980, Korea; silver21122@gmail.com; 3 SKKU Advanced Institute of Nanotechnology (SAINT), Sungkyunkwan University, Suwon 16419, Korea

**Keywords:** memristors, TiO_x_, stacking sequence, oxygen vacancy, resistive switching behaviour

## Abstract

The oxygen vacancies in the TiO_x_ active layer play the key role in determining the electrical characteristics of TiO_x_–based memristors such as resistive-switching behaviour. In this paper, we investigated the effect of a multi-layer stacking sequence of TiO_x_ active layers on the resistive-switching characteristics of memristor devices. In particular, the stacking sequence of the multi-layer TiO_x_ sub-layers, which have different oxygen contents, was varied. The optimal stacking sequence condition was confirmed by measuring the current–voltage characteristics, and also the retention test confirmed that the characteristics were maintained for more than 10,000 s. Finally, the simulation using the Modified National Institute of Standards and Technology handwriting recognition data set revealed that the multi-layer TiO_x_ memristors showed a learning accuracy of 89.18%, demonstrating the practical utilization of the multi-layer TiO_x_ memristors in artificial intelligence systems.

## 1. Introduction

Oxide-based memristive devices have attracted considerable interest due to their advantages such as non-volatile memory function, fast switching speed, low power consumption, good durability, process compatibility with complementary metal-oxide semiconductor technology, as well as the possibility of being implemented in real hardware and board-integrated systems [1,2,3,4,5,6]. In particular, the simple two-terminal crosspoint structure of memristors is expected to enable the high-density integration of computing devices by adopting three-dimensional stacking architectures [1,7]. Due to these advantages, various emerging electronics such as neuromorphic circuits and systems have been demonstrated by utilizing the memristors as one of their key elements [8,9]. From a conceptual point of view, the memristors are considered as the fourth fundamental circuit element in addition to resistors, capacitors, and inductors, which relate the charge *q* and the magnetic flux *φ* [10]. As an electronic device, on the other hand, the memristor behaves more like a memory device unit which stores the information in the form of resistance and changes according to the direction of the applied bias. Typically, the memristor devices are constructed with a metal−insulator−metal (MIM) structure with an active layer sandwiched between the two counter electrodes (bottom and top electrodes). Based on the history of the applied bias, the memristors are switched between high-resistive state (HRS) and low-resistive state (LRS) by the modulation of the resistance of the active layer. For the active layers, many different material candidates have been investigated such as TiO_2_, HfO_2_, NbO_2_, TaO_x_, ZnO and Al_2_O_3_ [11,12,13,14,15]. Also, for the formation of oxide active layers, deposition methods such as sputtering and anodizing have been utilized [16,17,18,19]. Among the candidate materials, TiO_2_- or TiO_2_/TiO_2-x_-based memristors have been intensively studied since the physical realization and understanding of the memristors in 2008 [20]. In TiO_2_- or TiO_2_/TiO_2−x_ memristors, the conducting state change is considered to originate in the formation and rupture of conducting channels [1,21]. As described by Carta et al., localized reduced TiO_x_ phases with lower O:Ti ratio are formed underneath the top electrode (TE) under an applied electric field [22]. Since the reduced TiO_x_ phases have a more metallic character than the stoichiometric TiO_2_ phase, the electrical conductivity is increased and LRS is obtained. Carta et al. also suggested that the reduction of the O:Ti ratio is involved with the migration of both O and Ti atoms that move toward the opposite directions under an applied bias [22]. In addition, atomic level simulations on the formation of conducting channels using kinetic Monte Carlo simulation have been reported [21,23,24]. In particular, according to the report from Li et al., it is claimed that filament formation is involved with the vacancy hopping induced localized electric field [21]. Based on these previous studies on the TiO_2_- and TiO_2_/TiO_2−x_-based memristors, it is likely that the oxygen vacancies play the key role in the operation of TiO_x_-based memristors.

Previously, various multilayer structure oxide memristors were investigated including TiO_2−x_/TiO_2_, TiON/HfO_y_/HfO_x_, TiO_2_/ZrO_2_, and TaO_x_/HfAl_y_O_x_ [25,26,27,28]. In this study, we constructed the active layer with a four-layer stacked structure of TiO_x_ films having different oxygen vacancy concentrations. Specifically, by varying the O_2_ partial pressure during the sputtering process of TiO_x_ films, TiO_x_ films with different oxygen-binding states could be obtained. Using these pre-defined deposition conditions, memristors with different stacking sequences are fabricated. To identify the role of the stacking sequence, we investigated the effect of stacking sequence on the memristive behaviours such as bistable switching characteristics, current on/off ratio, as well as their retention stability. Finally, for the practical demonstration of the fabricated device, we predicted the accuracy of Modified National Institute of Standards and Technology (MNIST) handwritten recognition by applying our device weight update characteristics.

## 2. Experimental Procedure

**Figure 1:**
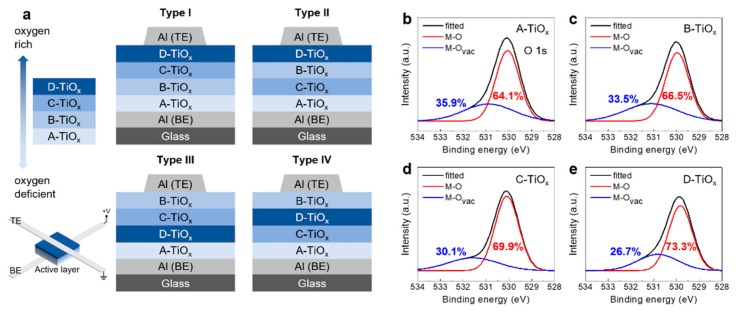
(**a**) Schematic device structures of the memristors having four TiO_x_ sub-layers with different oxygen vacancy (O_vac_) contents. The stacking sequence of the TiO_x_ sub-layers was varied (types I, II, III, and IV). The thicknesses of the A-TiO_x_, B-TiO_x_, C-TiO_x_, and D-TiO_x_ films were approximately 14.0 nm, 11.5 nm, 11.5 nm, and 9.0 nm, respectively. The O1s spectra measured by using the X-ray photoelectron spectroscopy (XPS) for, (**b**) A-TiO_x_, (**c**) B-TiO_x_, (**d**) C-TiO_x_, and (**e**) D-TiO_x_ films.

For the fabrication of TiO_x_-based memristor devices, a glass substrate was sonicated in acetone and isopropyl alcohol (IPA) for 10 min each. Then, the substrate was rinsed with IPA and dried with N_2_ gas. On the cleaned glass substrate, a 50 nm-thick Al electrode was deposited by thermal evaporation with a deposition rate of ~2 Å/s as a bottom electrode (BE). The patterning of the Al electrode was carried out by using a metal shadow mask and the width of the electrode was 50 μm. Next, for the deposition of multi-layer TiO_x_ active layers, a radio-frequency magnetron sputtering system was used with sputtering power and deposition pressure of 100 W and 5 × 10^−3^ Torr, respectively. To control the oxygen content in the TiO_x_ film, the argon (Ar) and oxygen (O_2_) gas flow rates were varied. Figure 1 shows the stacking sequence of the active layer. The patterning of TiO_x_ active layers was carried out by using a metal shadow mask which had a dimension of 1500 × 1500 μm. Finally, a 50 nm-thick Al top electrode was deposited by thermal evaporation and patterned by using a metal shadow mask. The width of the top electrode was 100 μm.

The atomic binding states of TiO_x_ films were analyzed by using X-ray photoelectron spectroscopy (XPS; Thermo Fisher Scientific, Waltham, MA, USA, ESCALAB 250). For the XPS analysis, each TiO_x_ sample was prepared separately. The current-voltage characteristic and the retention characteristics of the memristors were analyzed by using a semiconductor parameter analyzer (Agilent Technologies, Santa Clara, CA, USA, 4155C) which is attached to a probe station in dark ambient condition.

## 3. Results and Discussion

Figure 1a shows the schematic device structures of the memristors having four TiO_x_ sub-layers with different oxygen vacancy (O_vac_) contents. Here, the stacking sequence of the four TiO_x_ sub-layers was varied to find the structure to enhance the resistive switching characteristics of the device (types I, II, III, and IV). The four TiO_x_ sub-layers with different oxygen vacancy contents were fabricated by using different sputtering conditions by varying the gas flow rates of Ar and O_2_ gases (Ar:O_2_ = 50:5 sccm, 50:7 sccm, 50:10 sccm and 50:13 sccm). The corresponding TiO_x_ films are designated as A-TiO_x_, B-TiO_x_, C-TiO_x_, and D-TiO_x_, in the order of decreasing oxygen vacancy content (Figure 1a). Consequently, the A-TiO_x_ film is relatively oxygen-deficient, while the D-TiO_x_ film is relatively oxygen-rich. Also, the thicknesses of A-TiO_x_, B-TiO_x_, C-TiO_x_, and D-TiO_x_ films were approximately 14.0 nm, 11.5 nm, 11.5 nm, and 9.0 nm, respectively. To determine the variation of oxygen vacancy content, an XPS analysis was carried out. Figure 1b–e show the corresponding O1s spectra of the A-TiO_x_, B-TiO_x_, C-TiO_x_, and D-TiO_x_ films, respectively. Here, the fitted curve was deconvoluted to two main peaks centred at around ~530 eV and 531.0~531.5 eV. The peaks at ~530 eV and 531.0–531.5 eV represent the oxygen species in metal–oxygen–metal (M–O–M) and near the oxygen vacancy (O–M–O_vac_) [29], respectively. By increasing the P_O2_, the portion of oxygen vacancy was gradually decreased. For instance, in the case of A-TiO_x_ film, the portion of O–M–O_vac_ was 35.9%, while it decreased to 33.5%, 30.1%, and 26.7% for the B-TiO_x_, C-TiO_x_, and D-TiO_x_ film, respectively.

**Figure 2:**
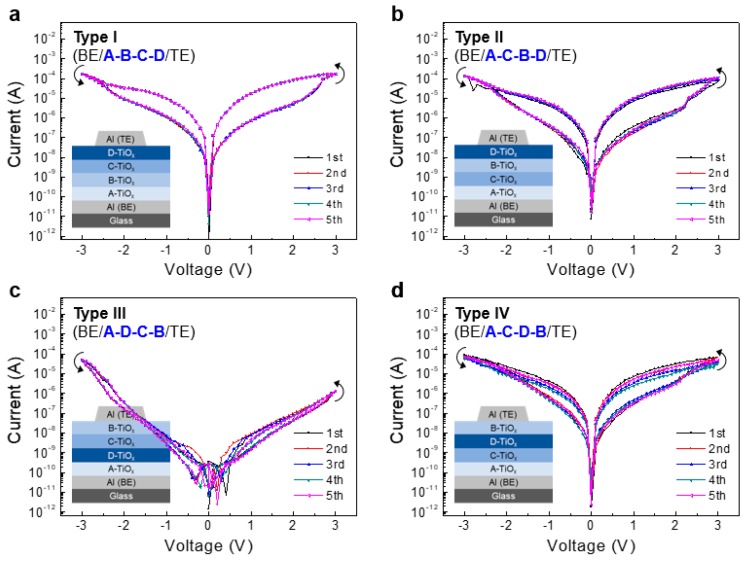
The representative current–voltage (I–V) characteristics of memristors having the stacking sequences of (**a**) A-B-C-D (type I), (**b**) A-C-B-D (type II), (**c**) A-D-C-B (type III), and (**d**) A-C-D-B (type IV), respectively. The top electrode (TE) was set as ground and the bias applied to the bottom electrode (BE) was swept in the sequence of 0 V → +3 V → −3 V → 0 V. The measurement was repeated five times in each case.

Since the oxygen vacancy concentration in an oxide film is strongly related to the electrical conductivity of the film [30] and the oxygen vacancies play the key role in the operation of TiO_x_-based memristors, it is likely that the stacking sequence of oxygen-rich and oxygen-deficient TiO_x_ films would influence the operation of the device. To investigate the effect of multi-layer stacking sequence on the resistive switching characteristics of the memristors, devices having four different stacking sequences were fabricated as schematically shown in Figure 1a. Figure 2a–d show the representative current-voltage (I–V) characteristics of the memristors having the stacking sequences of A-B-C-D (type I), A-C-B-D (type II), A-D-C-B (type III), and A-C-D-B (type IV), respectively. In all cases, the most oxygen-deficient layer, A-TiO_x_ film was placed in the bottom-most part, while the other layers were positioned with different combinations. Here, the TE electrode was set as ground and the bias applied to the BE was swept in the range of −3 V to +3 V to induce the SET and RESET processes. As indicated, the devices with types I, II, and IV stacking sequences showed memristive characteristics, clearly indicating the SET and RESET processes. In the meanwhile, the type III device showed only insulating behaviour without the bistable behaviour. In the case of the type II device (Figure 2b), the device first showed HRS (OFF state) upon sweeping the bias from 0 V to +3 V. Then, at around +2.3 V, transition to the LRS (ON state) starts to occur, which is regarded as the SET process. After reaching +3 V, the LRS maintains, exhibiting the memorizing behaviour. Then, by sweeping the bias to the negative direction, a transition from LRS to HRS occurs at around −2.7 V, which indicates the RESET process. Similar memristive characteristics and switching behaviours were also observed in devices with other stacking sequences such as types I and IV, with slightly different SET and RESET voltages. However, the I_ON_/I_OFF_ ratio was different depending on the device structure. In the tested device structures, the type II device (BE/A-C-B-D/TE) showed the highest I_ON_/I_OFF_ ratio value of ~45 (in average), while, the type I and type IV devices showed I_ON_/I_OFF_ ratio values of ~16 and ~17, respectively. The type III device (BE/A-D-C-B/TE), however, showed no switching behaviour and only insulating I–V characteristics were observed. Figure 2 also shows the I–V data which are repeated for five consecutive cycles. All the devices showed relatively stable I–V behaviour, while the type IV device showed a slight change in the current levels. As shown in Figure 2, during the SET and RESET processes, the current changes gradually, indicating the interface-type mechanism is dominant rather than the filament-type [31]. Concerning the variation of memristive behavior by the stacking sequence, we expect that the supply and migration of oxygen vacancies from the underneath TiO_x_ sub-layers to the TE/top-TiO_x_ interface are important [31]. In our results, the type I, II, and IV devices showed the memristive behaviour while the type III device showed insulating characteristics. In particular, in the cases of types I and II, the most oxygen-rich TiO_x_ layer (D-TiO_x_) with the lowest concentration of oxygen vacancies is placed on the top-most layer, contacting the TE, while the relatively oxygen-deficient TiO_x_ layers with larger concentrations of oxygen vacancies are placed underneath. Therefore, during the SET process, these relatively oxygen-deficient TiO_x_ layers can efficiently supply the oxygen vacancies and can contribute to the interface-type resistive switching behaviour. Comparing the type I and II devices, the positions of B- and C-TiO_x_ layers are different, where in type II, the second-most oxygen-deficient TiO_x_ layer (B-TiO_x_) is placed beneath the top D-TiO_x_ layer. Therefore, compared to type I device, more oxygen vacancies can be supplied to the top D-TiO_x_ layer, allowing larger resistive variation during switching. In the cases of types III and IV, the most oxygen-rich D-TiO_x_ layer is placed in the middle parts of the stacking. Therefore, the supply of oxygen vacancies towards the TE/top-TiO_x_ interface can be relatively smaller compared to type I and II devices. Also, considering that the oxygen vacancies migrate toward the TE/top-TiO_x_ interface during the SET process, the decrease of resistance in the D-TiO_x_ layer can be higher in type IV device compared to type III.

**Figure 3:**
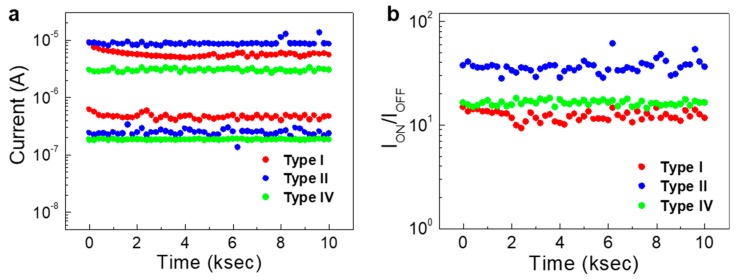
(**a**) The data retention characteristics of TiO_x_-based memristors (type I, II, and IV). The ON and OFF states were programmed with biases of +3 V and −3 V, respectively. Also, the read voltage was +1 V. (**b**) The variation of I_ON_/I_OFF_ ratio during the data retention test up to 10 ks.

The data retention characteristics of memristors are important for realizing highly stable memory devices as well as neuromorphic or synaptic devices. Figure 3a shows the data retention characteristics of type I, II, and IV devices. Here, the LRS and HRS states were programmed with pulsed biases of +3 V and −3 V, respectively (pulse widths of 100 ms). Also, the programmed states were read for an interval of 200 s up to 10,000 s, with a read voltage of +1 V. As displayed, devices with types I, II, and IV showed stable operation up to 10,000 s without a considerable change in the current levels of ON (*I*_ON_) and OFF (*I*_OFF_) states. Therefore, the *I*_ON_/*I*_OFF_ ratios are maintained correspondingly as shown in Figure 3b. Among the tested devices, the type II memristors exhibited the highest *I*_ON_/*I*_OFF_ ratio, while the type I memristor showed the lowest *I*_ON_/*I*_OFF_ ratio. Nonetheless, the results indicate that regardless of the stacking sequence, the TiO_x_-based memristors showed relatively stable operations.

**Figure 4:**
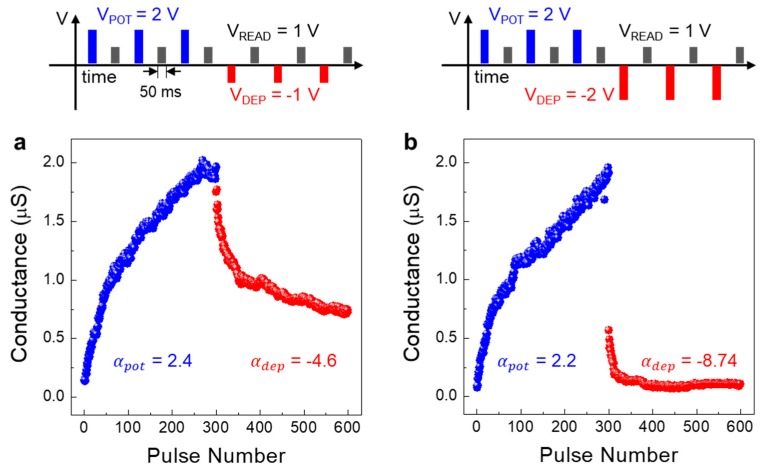
The weight update characteristics (conductance vs. number of pulse) of memristors with different depression pulse voltage (**a**) “A” pulse train condition (*V*_POT_ = +2 V, *V*_DEP_ = −1 V), and (**b**) “B” pulse train condition (*V*_POT_ = +2 V, *V*_DEP_ = −2 V).

Symmetric synaptic weight update characteristics between long-term potentiation (LTP) and long-term depression (LTD) is a crucial factor in designing synaptic devices that directly affects learning accuracy of neuromorphic computing [32,33]. As shown in Figure 4, we measured the long-term plasticity of the type II device by applying a pulse train consisting of potentiation (*V*_POT_), depression (*V*_DEP_) and read pulses. The V_POT_ was fixed at +2 V during the potentiation process while the *V*_DEP_ was varied as −1 V and −2 V as shown in Figure 4a (“A” pulse train) and b (“B” pulse train), respectively. Each potentiation and depression was performed for 300 cycles each. The duration time of each pulse was 50 ms and the pulse interval was ~1.45 s (see the inset of Figure 4 showing the pulse train of the three cycles). As shown in Figure 4, bidirectional switching behaviour was obtained in which the channel conductance was set to various conducting states between 0.136 μS and 2.02 μS for both conductance rise and fall processes. The acquired channel conductance represents a non-volatile behaviour. Thus, increasing and decreasing the channel conductance can be regarded as synaptic LTP and LTD, respectively. In addition to the symmetry, the change in linear conductance between LTP and LTD processes is also an important factor [32,33]. So, we calculated the nonlinearity values between LTP and LTD processes through the potentiation and depression data. The nonlinearity factors (α) were extracted from the characteristic curves shown in Figure 4. We use the device behavioural model [34], where the conductance change is represented with the following equations [34]:

(1) GLTP=B(1−e(PA))+Gmin

(2)   GLTD=B(1−e(P−PmaxA))+Gmax  

(3)   B=(GLTP−Gmin)∕(1−e(−PmaxA))  

where, P is number of pulses, B is a fitting parameter, A is the nonlinearity of potentiation ($\alpha_{pot}$) and depression ($\alpha_{dep}$), G_LTP_ and G_LTD_ are the conductance for LTP and LTD, and G_max_, G_min_ and P_max_ are the experimental data which represent the maximum conductance, minimum conductance and the maximum number of pulses required to change the device states between minimum and maximum conductance. The non-linearity values for “A” pulse train were $\alpha_{pot}\;$= 2.4 and $\alpha_{dep\;}\;$= −4.6 for the potentiation and depression, respectively (Figure 4a). The non-linearity values for “B” pulse train were $\alpha_{pot}\;$= 2.2 and $\alpha_{dep}\;$= −8.74 (Figure 4b).

**Figure 5:**
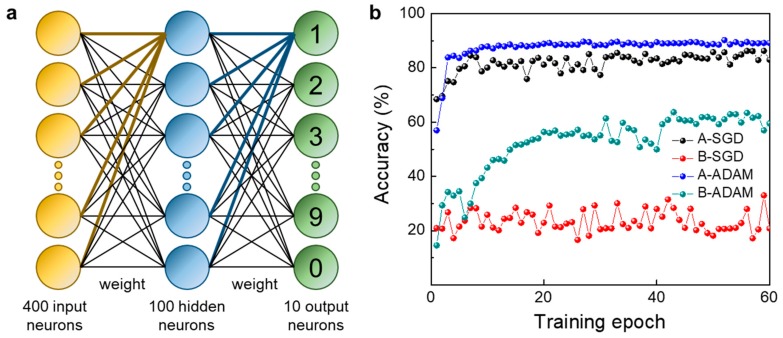
(**a**) A three-layer perceptron-based artificial neural network for Modified National Institute of Standards and Technology (MNIST) handwritten recognition, and (**b**) the accuracy with training epochs based on two pulse conditions and two algorithms which were stochastic gradient descent (SGD) and adaptive moment estimation (ADAM).

Using the “NeuroSim+” platform, a supervised artificial neural network learning simulation of the MNIST handwritten recognition data set was performed by applying the non-linearity, conductance level, and the cycle-to-cycle variation of our device [34,35]. In the simulation, we used a three-layer neural network with 400 pre-neurons, 100 hidden neurons and 10 output neurons which correspond to 10 classes of digits (0–9). The detail of the three-layer multilayer perceptron network is shown in Figure 5a [36]. The TiO_x_ multi-layer memristor acts as a memory element in a crossbar array and their memristor conductivity change was used as the weight update to run the back-propagation algorithm. Here, the crossbar is considered as part of a “neuron core” that executes vector-matrix multiplication (inference) and outer-product updates (learning) operations [37]. The sum of the input neuron signal vector and the first layer of the synaptic weight is transferred to the input vector of the hidden layer after activation and binarization [9]. For each epoch, 60,000 training data set were used for training, and accuracy was obtained using a 10,000 test data set.

By using algorithmic methods which was stochastic gradient descent (SGD) and adaptive moment estimation (ADAM) weight update, the accuracy of MNIST handwritten recognition was obtained. As shown in Figure 5b, in the case of the “A” pulse train condition (*V*_POT_ = +2 V, *V*_DEP_ = −1 V), the accuracy was 82.99% when using the SGD, and 89.18% when using the ADAM. On the other hand, in the case of ‘B’ pulse train condition (*V*_POT_ = +2 V, *V*_DEP_ = −2 V), the accuracy was 20.80% when using the SGD, and 59.52% when using the ADAM. Because the LTD non-linearity value of the “A” pulse train condition is smaller compared to that of the “B” pulse train condition, it is advantageous for learning process which resulted in a higher accuracy. The learning algorithm also plays an important role in accuracy. The SGD method calculates an error from the current weight, predicting a direction in which the weight should change, and learns at a predetermined step size. However, the ADAM method stored the exponential average of the slopes calculated so far to modulate the weight direction, and also stored the exponent average of the squared slopes to set the step size [38,39]. Thus, when using the ADAM method, there were more data to store, but accurate learning could be achieved using our device.

## 4. Conclusions

In this paper, we demonstrated the multi-layer TiO_x_ memristors for potential memory and neuromorphic applications. The four TiO_x_ sub-layers with different oxygen vacancy content were fabricated by using different sputtering conditions by varying the gas flow rates of Ar and O_2_. Also, through the XPS analysis, the ratio of M–O–M and M–O_vac_ of oxygen peaks at different gas flow conditions was determined. By comparing the memristor characteristics according to the stacking sequence of the memristor device with a multi-layer structure, it was confirmed that the I_ON_/I_OFF_ ratio value is the highest as 45 in the type II stacking sequence structure. Also, by measuring the retention time in the on state and off state, it was confirmed that the current is maintained without degradation over 10,000 s. In addition, the long-term plasticity (LTP/LTD) was measured for the type II stacking sequence structure to obtain LTP and LTD non-linearity according to different depression voltage pulses. Two depression pulse voltage conditions and algorithm methods (SGD and ADAM) were used, and the highest accuracy of 89.18% was obtained when *V*_DEP_ = −1 V and with the ADAM algorithm.
